# Efficacy and Safety of Percutaneous Kyphoplasty Combined With Zoledronic Acid for Osteoporotic Vertebral Compression Fractures: A Systematic Review and Meta-Analysis

**DOI:** 10.7759/cureus.98776

**Published:** 2025-12-08

**Authors:** Muhammad Tayyab, Mahmood Ahmad, Rizwan Akbar, Rahman Syed, Khwaja Irfan Ullah, Naeem Ul Haq, Imran Khan, Ameer Afzal Khan, Anfal Khan, Muhammad Hassaan Javaid

**Affiliations:** 1 Trauma and Orthopaedics, Hayatabad Medical Complex Peshawar, Peshawar, PAK; 2 Truama and Orthopaedics, Milton Keynes University Hospital, Milton Keynes, GBR; 3 Trauma and Orthopaedics, University College Hospital, London, GBR; 4 Internal Medicine, Swat Medical College, Saidu Sharif, PAK; 5 Neurosurgery, Saidu Teaching Hospital, Saidu Sharif, PAK; 6 Neurosurgery, Bacha Khan Medical College, Mardan, PAK; 7 General Surgery, Saidu Teaching Hospital, Saidu Sharif, PAK; 8 Internal Medicine, Saidu Medical College, Saidu Sharif, PAK; 9 Internal Medicine, Shifa College of Medicine, Islamabad, PAK

**Keywords:** bilateral percutaneous kyphoplasty, osteoporotic vertebral fractures, systematic review and meta-analysis, unilateral percutaneous curved kyphoplasty, zoledronic acid hydrate

## Abstract

Osteoporotic vertebral compression fractures (OVCFs) are a significant source of pain and disability among the elderly. While percutaneous kyphoplasty (PKP) offers mechanical stabilization and pain relief, it does not address the underlying bone fragility. Zoledronic acid (ZOL), a strong bisphosphonate, makes bones stronger and may stop them from breaking again. This meta-analysis assessed the efficacy and safety of combining PKP with ZOL versus PKP alone in patients with OVCFs. A systematic search of PubMed, Cochrane Library, and Clinicaltrials.gov was performed up to August 2025 following Preferred Reporting Items for Systematic Reviews and Meta-Analyses (PRISMA) guidelines. Randomized controlled trials (RCTs) comparing PKP plus ZOL versus PKP alone were included. Data were pooled using Review Manager (RevMan) version 5.4 (The Cochrane Collaboration, London, England, UK). Mean difference (MD) and risk ratios (RRs) with 95% confidence intervals (CIs) were calculated using random-effects models. Five RCTs involving 997 patients (499 PKP + ZOL; 498 PKP) were analyzed. The combination therapy showed greater pain reduction (visual analog scale (VAS): MD = -1.18 (-2.04, -0.31)), improved function (Oswestry Disability Index (ODI): MD = -9.18 (-21.57, 3.20)), and increased Cobb angle correction (MD = 1.88 (0.14, 3.63)). Bone mineral density (BMD) improved significantly (MD = 0.08 (0.06, 0.11)), while bone turnover markers (procollagen type I N-terminal propeptide (PINP), β-C-terminal telopeptide (β-CTX), and N-terminal mid-fragment of osteocalcin (N-MID osteocalcin)) were markedly reduced. Adverse events were more common with ZOL (RR = 5.46 (3.75, 7.96)) but were mild and transient. In OVCF patients, combining PKP with ZOL gives better clinical, functional, and bone-strengthening advantages than PKP alone. Even while small side effects happen more often, the treatment is still safe and well-tolerated. This combined method works well to fix both the mechanical stability and the metabolic fragility of osteoporotic fractures.

## Introduction and background

Osteoporotic vertebral compression fractures (OVCFs) are among the most common and disabling complications of osteoporosis, particularly in the elderly, leading to chronic pain, reduced mobility, kyphotic deformities, and a profound decline in quality of life [1,2]. Standard management typically includes conservative measures such as analgesics, bracing, and anti-osteoporotic medications. While these strategies may provide symptom relief, they often fail to restore vertebral structure or prevent subsequent fractures, leaving patients at high risk for recurrent injury and long-term disability [3].

Percutaneous kyphoplasty (PKP), a minimally invasive vertebral augmentation procedure, has emerged as the recommended treatment option for OVCFs. It quickly relieves pain, restores vertebral body height, and corrects spinal deformity by anchoring the fractured vertebra using bone cement [4]. However, PKP alone does not address the underlying osteoporotic process, leaving patients at risk for new or adjacent vertebral fractures [5].

Zoledronic acid (ZOL), a potent nitrogen-containing bisphosphonate, inhibits osteoclast-mediated bone resorption and has been shown to improve bone mineral density (BMD) and lower fracture risk [6]. Recent research has looked into the combination of PKP and ZOL, arguing that ZOL can supplement the mechanical effects of PKP by enhancing bone quality and lowering the risk of future fractures [7,8]. Nonetheless, evidence for the combination's cumulative clinical and radiological benefits is mixed. Some studies found that combination therapy provided better pain relief, functional recovery, and vertebral repair than PKP alone [9,10], whilst others found no significant advantage [11].

Given the growing number of studies on this topic, a systematic synthesis of available evidence was considered essential to clarify the efficacy and safety of PKP combined with ZOL. This meta-analysis aimed to determine whether combination therapy is superior to PKP alone in terms of pain relief, functional improvement, vertebral height restoration, bone metabolism, and adverse events in patients with OVCFs.

## Review

Methodology

Study Design and Protocol Registration

This study was conducted according to the Preferred Reporting Items for Systematic Reviews and Meta-Analyses (PRISMA) 2020 guidelines [12]. The protocol for this systematic review and meta-analysis was prospectively registered in the International Prospective Register of Systematic Reviews (PROSPERO) [13]. *Eligibility Criteria*

Studies were considered eligible if they met the following criteria: randomized controlled trials (RCTs) that were carried out on adult patients (≥ 50 years) with radiologically confirmed OVCFs. The intervention group received a combination of PKP and ZOL, while the control group received PKP alone or ZOL alone. Studies were excluded if they were non-randomized, featured fractures caused by cancer, trauma, or infection, or lacked sufficient quantitative data for analysis.

Search Strategy

A comprehensive literature search was conducted in PubMed, Cochrane Library, and Clinicaltrials.gov from inception to August 2025. The following keywords and Medical Subject Headings (MeSH) terms were used in various combinations: “percutaneous kyphoplasty”, “zoledronic acid”, “osteoporotic vertebral compression fracture”, “osteoporosis”, “vertebral height”, and “pain”.

Boolean operators (AND, OR) were applied to refine the search. Additionally, the reference lists of eligible articles were manually screened to identify any relevant studies not captured by the database search. No language restrictions were applied. *Study Selection*

All retrieved titles and abstracts were screened by two independent reviewers. The full texts of potentially relevant studies were reviewed for eligibility based on the inclusion criteria. Discrepancies were resolved by discussing or consulting with a third reviewer. The study selection process was summarized using a PRISMA flow diagram. *Data Extraction*

Data were extracted using a standardized form that included study characteristics (author, year, sample size, design), participant demographics, intervention details, follow-up duration, and reported outcomes. Any discrepancies were resolved through discussion or consultation with a third reviewer. *Outcome Measures*

The primary outcomes of interest were changes in the visual analog scale (VAS) [14], Oswestry Disability Index (ODI) [15], Cobb angle [16], and vertebral height restoration. Secondary outcomes included changes in BMD [17] of the proximal femur, bone turnover markers (procollagen type I N-terminal propeptide (PINP), β-C-terminal telopeptide (β-CTX), and N-terminal mid-fragment of osteocalcin (N-MID osteocalcin)) [18], and the incidence of adverse events such as cement leakage, myalgia, arthralgia, pyrexia, and influenza-like symptoms.

Quality Assessment

The methodological quality of included RCTs was evaluated using the Cochrane Risk of Bias 2.0 (RoB-2) tool [19]. Each study was assessed across domains, including randomization, allocation concealment, blinding, incomplete outcome data, and selective reporting. The overall risk of bias was categorized as low, some concerns, or high.

Statistical Analysis

Data synthesis was performed using Review Manager (RevMan) version 5.4 (The Cochrane Collaboration, London, England, UK) [20]. For continuous outcomes such as VAS score [14], ODI [15], Cobb angle [16], and BMD [17], results were expressed as mean difference (MD) with 95% confidence intervals (CIs). For dichotomous outcomes (e.g., adverse events), risk ratios (RRs) with 95% CIs were calculated.

Heterogeneity was assessed using the I² statistic, with I² > 50% indicating substantial heterogeneity. A random-effects model (DerSimonian-Laird method) was applied when heterogeneity was significant; otherwise, a fixed-effects model was used. Subgroup analyses were performed based on follow-up duration (six months vs. 12 months) for VAS and ODI outcomes.

Results

**Figure 1:**
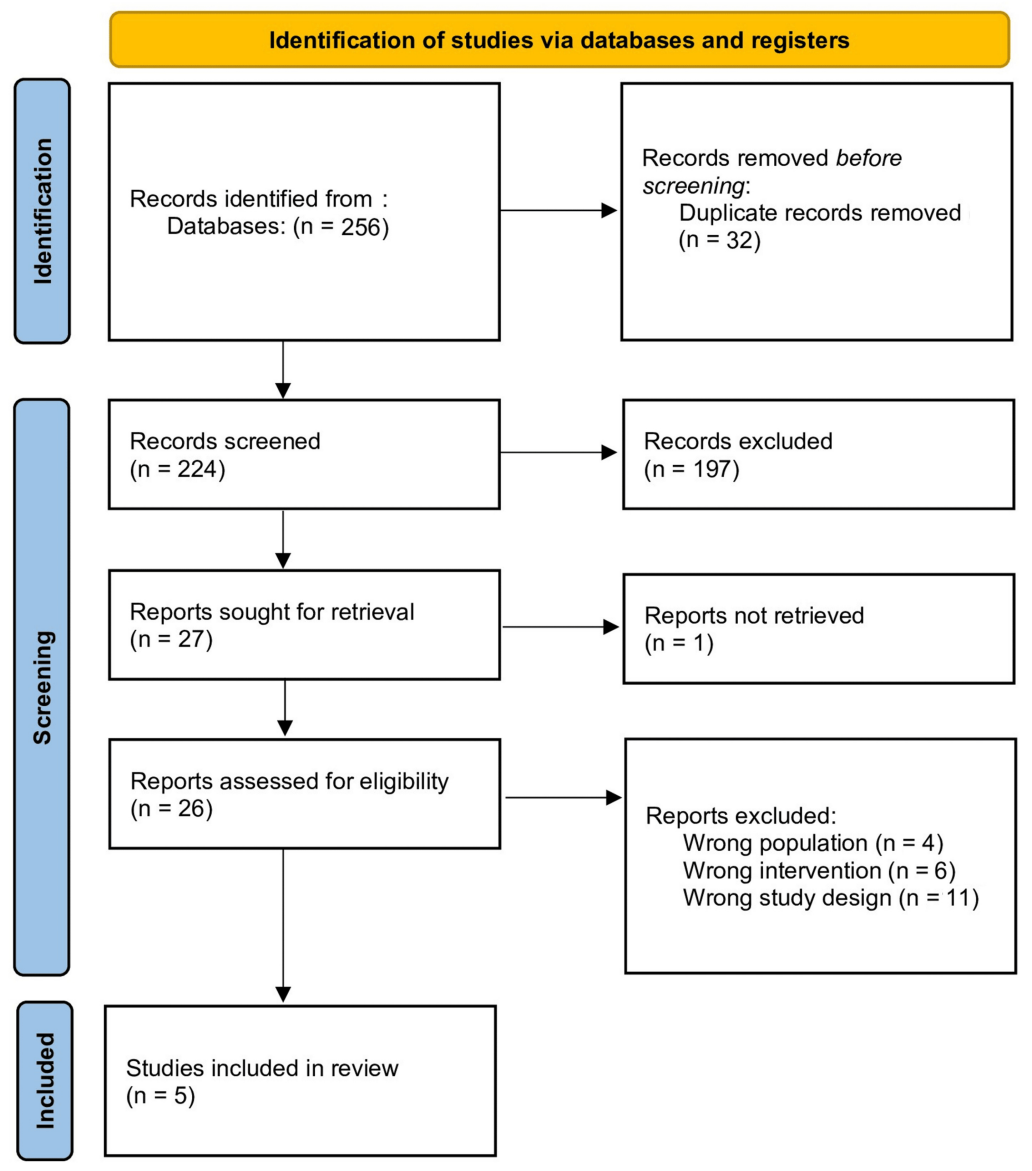
PRISMA flowchart of study selection PRISMA, Preferred Reporting Items for Systematic Reviews and Meta-Analyses [12]

The initial search identified 256 studies, of which 27 were screened in full text after title and abstract review. Finally, five RCTs [21-25] comprising 997 participants (499 in the combination group and 498 in the control group) met the inclusion criteria and were included in the meta-analysis, as shown in the PRISMA flowchart (Figure 1).

The included studies were published between 2017 and 2024, with follow-up durations ranging from six months to three years. All studies compared PKP combined with ZOL against PKP alone. The main characteristics of the included trials are summarized in Table 1.

**Table 1:** Characteristics of the included studies PKP, percutaneous kyphoplasty; ZOL, zoledronic acid; VAS, visual analog scale; ODI, Oswestry Disability Index; BMD, bone mineral density; AE, adverse events; VC, vertebral compression; ORR, overall response rate; BAP, bone alkaline phosphatase; BGP, bone Gla protein; CTX, C-terminal telopeptide; PINP, procollagen type I N-terminal propeptide; ADL, activities of daily living

Author and Year	Design	Intervention (I)	Control (C)	Sample	Outcomes	Follow-up
Hongchi Yi et al., 2024 [21]	RCT	PKP + ZOL	ZOL/PKP	I = 200, C = 200	VAS, ODI, ADL, height of VC, kyphosis angle, ORR, BAP, BGP, CTX	1 month, 1 year
Bin Liu et al., 2018 [22]	RCT	PKP + ZOL	PKP	I = 52, C = 52	Clinical efficacy, VAS, ODI, BMD	1 year
K. Lu et al., 2021 [23]	RCT	PKP + ZOL	PKP	I = 78, C = 76	VAS, ODI, AE, mean compression, height lost	1 year, 2 years, 3 years
Kan Liu et al., 2023 [24]	RCT	PKP + ZOL	PKP	I = 119, C = 119	VAS, ODI, BMD, Cobb angle, vertebrae height, AE	6 months, 12 months
J. Zhang et al., 2019 [25]	RCT	PKP + ZOL	PKP	I = 50, C = 51	VAS, BMD, recompression fracture, AE	6 months, 1 year

Pain Relief (VAS)

**Figure 2:**
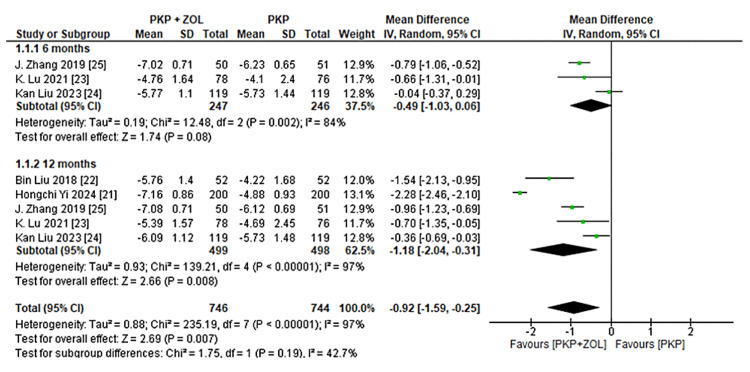
Forest plot of the mean difference in VAS scores between patients treated with PKP + ZOL and PKP alone at six and 12 months PKP, percutaneous kyphoplasty; ZOL, zoledronic acid; CI, confidence interval; SD, standard deviation; VAS, visual analog scale [14,21-25]

All five studies reported changes in pain scores using the VAS [14]. The pooled analysis demonstrated a greater reduction in VAS scores in the PKP plus ZOL group compared to the PKP alone group. At six months, the MD was -0.49 (-1.03, 0.06), and at 12 months, the MD was -1.18 (-2.04, -0.31), favoring combination therapy, as shown in Figure 2. However, substantial heterogeneity was observed across studies (I² > 75%), likely due to differences in baseline severity and follow-up intervals.

Functional Improvement (ODI)

**Figure 3:**
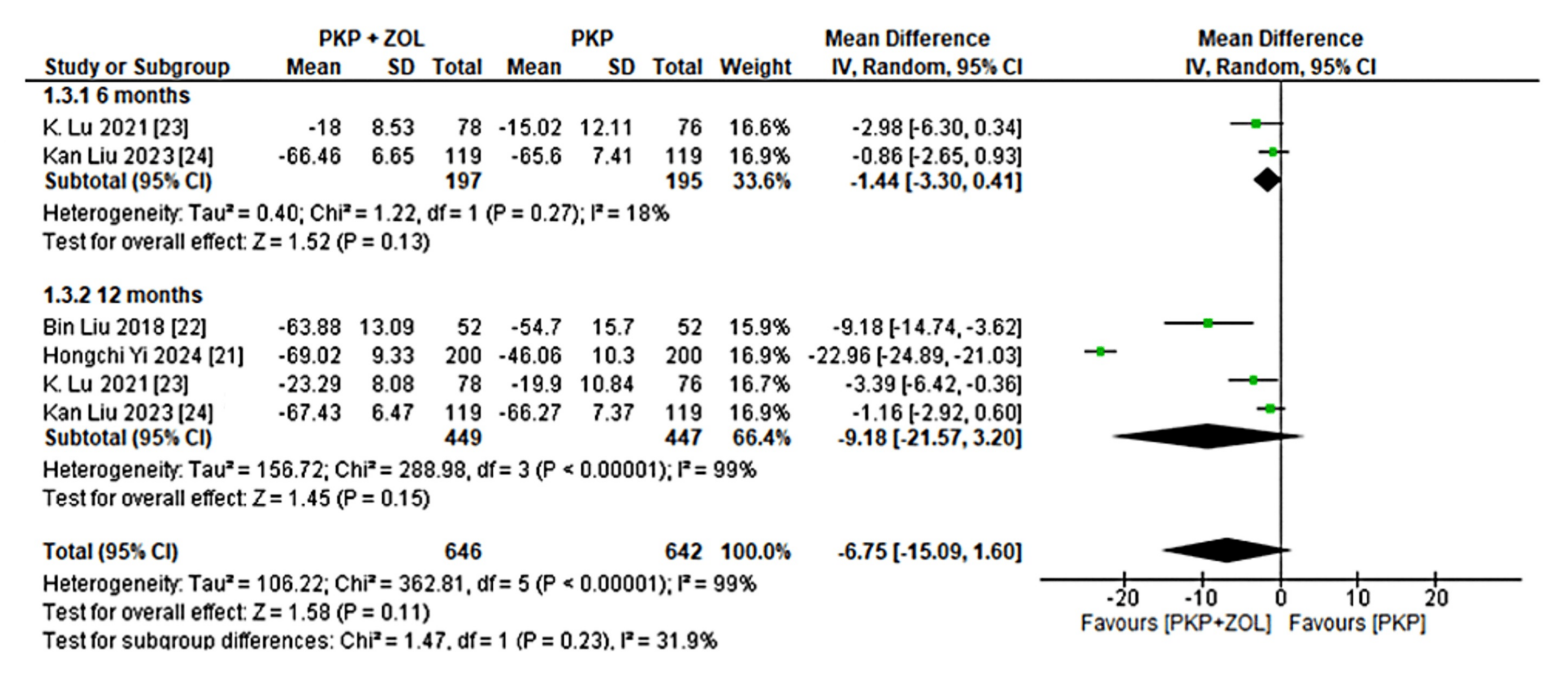
Forest plot of ODI improvement in patients treated with PKP + ZOL versus PKP alone PKP, percutaneous kyphoplasty; ZOL, zoledronic acid; CI, confidence interval; SD, standard deviation; ODI, Oswestry Disability Index [15,21-24]

Four studies reported ODI [15] scores. A pooled analysis revealed that patients receiving PKP combined with ZOL had better functional outcomes than those receiving PKP alone. The mean difference in ODI was -1.44 (-3.30, 0.41) at six months and -9.18 (-21.57, 3.20) at 12 months, as shown in Figure 3. Although the trend favored the combination therapy, results were limited by high heterogeneity.

Radiological Outcomes (Cobb Angle and Vertebral Height)

**Figure 4:**
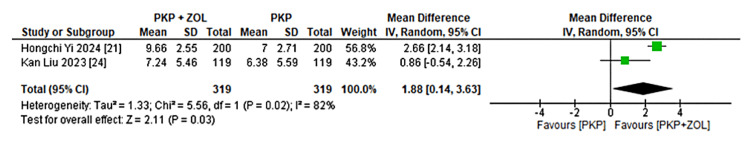
Forest plot showing the change in Cobb angle following treatment with PKP + ZOL versus PKP alone PKP, percutaneous kyphoplasty; ZOL, zoledronic acid; CI, confidence interval; SD, standard deviation [16,21,24]

Three studies assessed radiographic parameters. The Cobb angle [16] was significantly improved in the combination group, with a pooled mean increase of 1.88° (0.14, 3.63) compared to PKP alone, as shown in Figure 4. Improvement in vertebral height was consistently greater among patients receiving ZOL, though variations in measurement methods precluded quantitative pooling.

BMD

**Figure 5:**
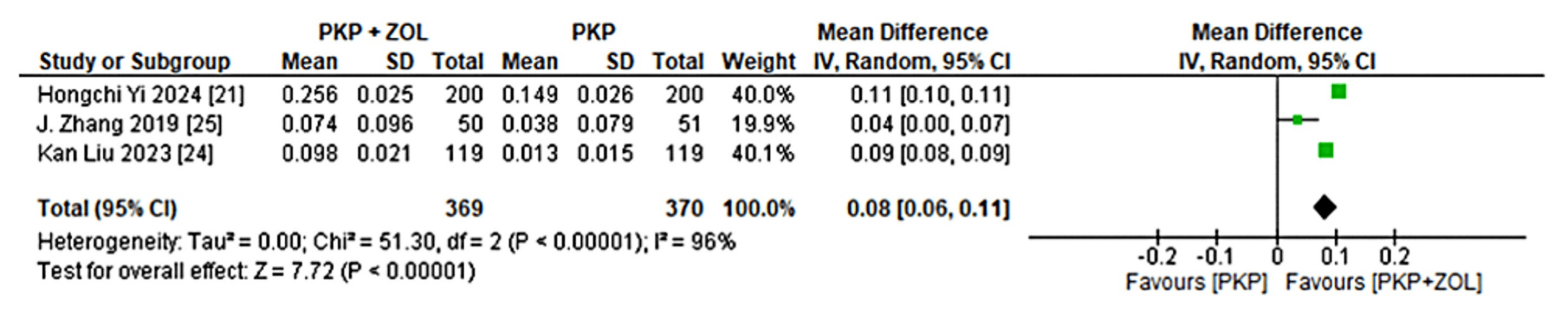
Forest plot of the mean change in BMD at the proximal femur between patients treated with PKP + ZOL and PKP alone PKP, percutaneous kyphoplasty; ZOL, zoledronic acid; CI, confidence interval; SD, standard deviation; BMD, bone mineral density [17,21,24,25]

Three trials reported changes in BMD [17] at the proximal femur after 12 months. Meta-analysis showed a significant improvement in the combination group, with an MD of 0.08 (0.06, 0.11) compared to PKP alone, as shown in Figure 5, indicating enhanced bone strength with ZOL therapy.

Bone Metabolism Markers

**Figure 6:**
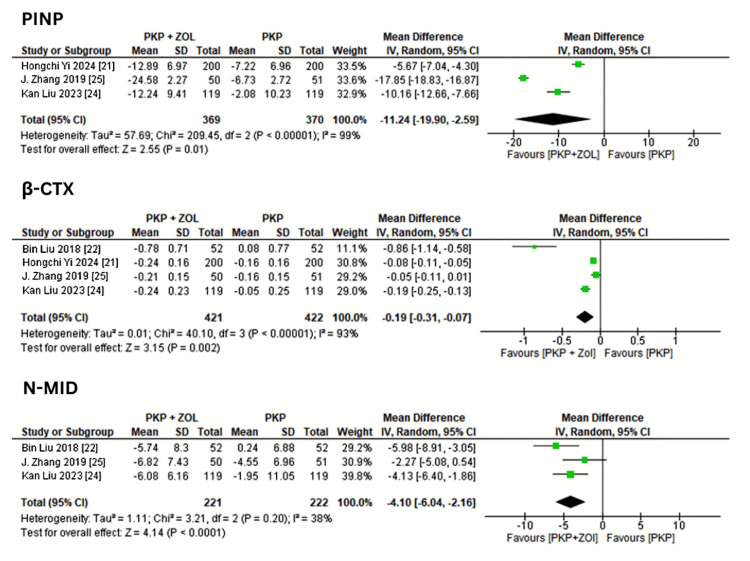
Forest plot demonstrating reduction in bone metabolism markers following treatment with PKP + ZOL versus PKP alone PKP, percutaneous kyphoplasty; ZOL, zoledronic acid; CI, confidence interval; SD, standard deviation; β-CTX, β-C-terminal telopeptide; PINP, procollagen type I N-terminal propeptide; N-MID osteocalcin, N-terminal mid-fragment of osteocalcin [18,21,22,24,25]

Two studies measured bone turnover markers. The pooled data demonstrated significant reductions in PINP (MD = -11.24 (-19.90, -2.59)), β-CTX (MD = -0.19 (-0.31, -0.07)), and N-MID osteocalcin (MD = -4.10 (-6.04, -2.16)) in the combination group compared to controls, as shown in Figure 6.

Adverse Events

Three studies reported adverse events, as shown in Table 2. The overall incidence of any adverse event was significantly higher in the combination group (57.9%) compared to the control group (10.6%) (RR = 5.46 (3.75, 7.96)). The most common events were pyrexia (RR = 53.82 (13.53, 214.12)), myalgia (RR = 24.89 (3.41, 181.53)), and arthralgia (RR = 20.91 (2.83, 154.68)), which are known transient side effects of ZOL. However, there were no significant differences in cement leakage rates between groups (RR = 1.14 (0.69, 1.88)). No serious adverse outcomes such as renal failure or osteonecrosis of the jaw were reported.

**Table 2:** Comparison of adverse events between patients treated with PKP + ZOL and PKP alone PKP, percutaneous kyphoplasty; ZOL, zoledronic acid; CI, confidence interval; RVF, recompression vertebral fracture

Adverse Event	Intervention Group	Control Group	Risk Ratio	95% CI	Studies (n)
Total patients	247	246	-	-	3
Any adverse event	143/247 (57.9%)	26/246 (10.6%)	5.46	(3.75, 7.96)	3
Pyrexia/fever	108/247 (43.7%)	2/246 (0.8%)	53.82	(13.53, 214.12)	3
Cement leakage	30/197 (15.2%)	26/195 (13.3%)	1.14	(0.69, 1.88)	2
Myalgia	25/247 (10.1%)	1/246 (0.4%)	24.89	(3.41, 181.53)	3
Arthralgia	21/247 (8.5%)	1/246 (0.4%)	20.91	(2.83, 154.68)	3
Influenza-like symptoms	25/197 (12.7%)	0/195 (0%)	∞	(∞, ∞)	2
Headache	9/197 (4.6%)	1/195 (0.5%)	8.91	(1.14, 69.45)	1
RVF	0/50 (0%)	5/51 (9.8%)	0	(0.00, 0.00)	1

Risk of Bias

**Figure 7:**
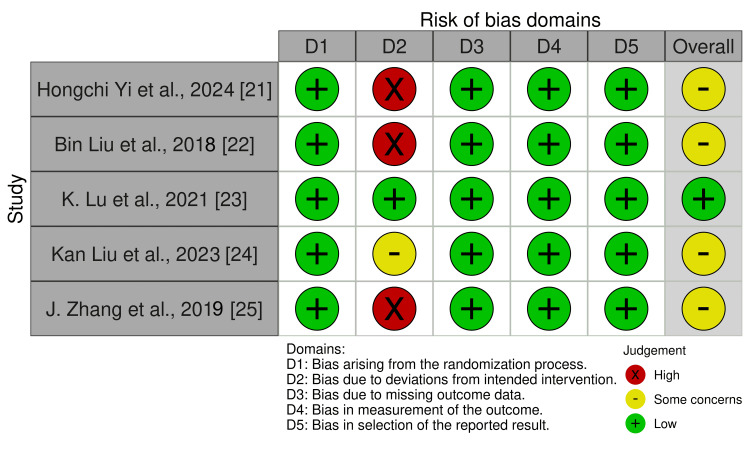
Risk of bias assessment of the included RCTs using the Cochrane Risk of Bias 2.0 (RoB-2) tool RCT, randomized controlled trials [19,21-25]

All five RCTs had a low risk of randomization and reporting, as shown in Figure 7. Three studies had a high risk due to a lack of blinding. Overall, one study was low risk [23], and four studies had some concerns [21,22,24,25]. 

Discussion

This systematic review and meta-analysis assessed the efficacy and safety of combining PKP with ZOL in the treatment of OVCFs. Based on data from five RCTs involving 997 patients, the findings show that the combination of PKP and ZOL provides better long-term pain relief, functional improvement, vertebral restoration, and bone metabolism control than PKP alone, although with a higher incidence of mild, transient adverse effects.

Pain reduction and functional recovery are the main goals of OVCF treatment. At both six and 12 months, our pooled results showed that patients receiving PKP plus ZOL had significantly lower VAS and ODI ratings than patients getting PKP alone. These findings are consistent with prior research indicating that PKP provides immediate mechanical stabilization and pain relief, whereas ZOL improves analgesic and structural advantages by slowing osteoclastic bone resorption [4,26]. The observed improvements in functional outcomes may also be related to the maintenance of vertebral height and the prevention of progressive kyphosis, which are strongly correlated with better quality of life in OVCF patients [27]. Radiological measures, such as vertebral height and Cobb angle, improved significantly in the combination group. The mean Cobb angle rise of 1.88° indicates that ZOL may help to maintain spinal alignment over time. ZOL inhibits bone turnover, enhances microarchitectural repair, and alleviates mechanical stress on surrounding vertebrae [6]. Previous studies have found that administering ZOL following vertebral augmentation lowers the incidence of new or nearby fractures, improving overall spine stability [8,25]. This could account for the radiological superiority seen in our analysis. This combination therapy significantly increased BMD while decreasing bone turnover markers, such as PINP, β-CTX, and N-MID osteocalcin. These data corroborate ZOL's anti-resorptive effects, which are consistent with previous studies showing continuous suppression of bone remodeling and improved BMD in osteoporotic populations [18,28]. Improved bone quality after ZOL therapy is likely to help minimize refractures following PKP, which has been a significant limitation of surgical-only methods [8]. The combination group experienced more adverse effects, primarily temporary pyrexia, myalgia, arthralgia, and flu-like symptoms, which are typical acute-phase reactions to intravenous bisphosphonates [29]. However, these effects were self-limiting, and no major problems, such as renal impairment or jaw osteonecrosis, were seen. Importantly, there was no significant difference in cement leakage rates between groups, demonstrating that ZOL does not raise procedural risk. The present findings indicate that PKP coupled with ZOL provides both mechanical and metabolic benefits. While PKP quickly repairs vertebral structure, ZOL preserves bone mass and lowers the likelihood of recurrent fractures. This dual-action approach addresses both the symptomatic and pathophysiological aspects of OVCF, making it a potentially superior management strategy [10,21]. Furthermore, long-term gains in BMD and vertebral integrity may result in lower healthcare expenses and greater patient independence. Despite the promising results, several limitations should be taken into account. First, the number of included studies was limited, and most had moderate sample sizes, which may restrict generalizability. Second, substantial heterogeneity was observed in several pooled analyses, likely due to variations in follow-up duration, baseline fracture severity, and dosing protocols for ZOL. Third, all included trials focused on PKP, limiting direct comparisons with vertebroplasty or spinal fusion in combination with osteoclastic agents. Fourth, publication bias could not be formally assessed due to the small number of trials. Fifth, long-term outcomes beyond three years remain underexplored, and there is insufficient data on fracture recurrence and quality of life measures over extended follow-up. Finally, differences in outcome measurement methods across studies, particularly for radiographic parameters, may have affected pooled estimates. Future multicenter RCTs with standardized protocols and longer follow-up are warranted to address these gaps.

## Conclusions

This meta-analysis demonstrated that the combination of PKP and ZOL offers superior clinical and radiological outcomes compared to PKP alone in patients with OVCFs. The inclusion of ZOL significantly improved long-term pain alleviation, functional recovery, vertebral height restoration, and BMD while decreasing bone turnover markers, indicating increased bone metabolism and strength. Although the combination therapy was linked to a higher prevalence of temporary adverse reactions such as fever, myalgia, and arthralgia, these side effects were minor and self-limiting, with no major consequences noted. Overall, PKP combined with ZOL addresses both the mechanical instability and metabolic fragility that cause osteoporotic vertebral fractures, resulting in a more holistic treatment strategy. Future large-scale, multicenter randomized trials with extended follow-up periods are needed to validate these findings, investigate appropriate dose regimes, and evaluate long-term fracture prevention and safety outcomes.
